# Temporal trends and outcomes of heart transplantation in Spain (2002–2021): propensity score matching analysis to compare patients with and without type 2 diabetes

**DOI:** 10.1186/s12933-023-01995-1

**Published:** 2023-09-29

**Authors:** Ana Lopez-de-Andres, Rodrigo Jiménez-García, Valentin Hernández-Barrera, David Carabantes-Alarcon, Jose J. Zamorano-Leon, Ricardo Omaña Palanco, Jose L. del-Barrio, Javier de-Miguel-Díez, Jose M. de-Miguel-Yanes, Natividad Cuadrado-Corrales

**Affiliations:** 1 Department of Public Health & Maternal and Child Health, Faculty of Medicine, Universidad Complutense de Madrid, Madrid, 28040 Spain; 2 Preventive Medicine and Public Health Teaching and Research Unit. Health Sciences Faculty, Rey Juan Carlos University, Alcorcón, Madrid, Spain; 3 Respiratory Care Department, Hospital General Universitario Gregorio Marañón, Universidad Complutense de Madrid, Instituto de Investigación Sanitaria Gregorio Marañón (IiSGM), Madrid, Spain; 4 Internal Medicine Department, Hospital General Universitario Gregorio Marañón, Universidad Complutense de Madrid, Instituto de Investigación Sanitaria Gregorio Marañón (IiSGM), Madrid, Spain

**Keywords:** Type 2 diabetes, Heart transplantation, Hospitalizations, Propensity score matching, Discharge database.

## Abstract

**Background:**

The impact of Type 2 Diabetes (T2D) on the outcomes of heart transplantation (HT) has not yet been clearly established. The objectives of this study were to examine the trends in the prevalence of T2D among individuals who underwent a HT in Spain from 2002 to 2021, and to compare the clinical characteristics and hospitalization outcomes between HT recipients with and without T2D.

**Methods:**

We used the national hospital discharge database to select HT recipients aged 35 and older. The International Classification of Diseases, Ninth and Tenth Revisions (ICD-9 and ICD-10) were used to identify patients with and without T2D. We also recorded comorbidities, complications of HT, and procedures. Propensity score matching (PSM) and Cox regression were used to analyze the effect of T2D on in-hospital mortality (IHM).

**Results:**

Between 2002 and 2021, a total of 4429 HTs (T2D, 19.14%) were performed in Spain. The number of HTs in patients with T2D decreased from 2002 to 2005 (n = 171) to 2014–2017 (n = 154), then rose during 2018–2021 (n = 186). Complications of HT increased in patients with and without T2D over the study period (26.9% and 31.31% in 2002–2005 vs. 42.47% and 45.01% in 2018–2021, respectively). The results of the PSM showed that pneumonia and Gram-negative bacterial infections were less frequent in patients with T2D and that these patients less frequently required hemodialysis, extracorporeal membrane oxygenation (ECMO), and tracheostomy. They also had a shorter hospital stay and lower IHM than patients without diabetes. The variables associated with IHM in patients with T2D were hemodialysis and ECMO. IHM decreased over time in people with and without T2D. The Cox regression analysis showed that T2D was associated with lower IHM (HR 0.77; 95% CI 0.63–0.98).

**Conclusions:**

The number of HTs increased in the period 2018–2021 compared with 2002–2005 in patients with and without T2D. Over time, complications of HT increased in both groups studied, whereas IHM decreased. The presence of T2D is associated with lower IHM.

**Supplementary Information:**

The online version contains supplementary material available at 10.1186/s12933-023-01995-1.

## Background

Type 2 diabetes (T2D) frequently affects patients with advanced heart failure, often concomitantly with other medical conditions such as obesity, thromboembolic complications, renal dysfunction, and immunosuppression, thereby increasing the risk of infection [1, 2]. Accordingly, given that heart transplantation (HT) is an alternative in advanced heart failure, T2D is deemed a risk factor that could potentially influence its outcome [3].

No consensus has been reached with respect to the impact of T2D on complications after HT: some studies report no significant increase in the risk of infection, rejection, or heightened post-transplant complications in recipients with T2D [4–6], whereas others have suggested the contrary [7–9]. These inconsistencies may be due to variations in study design, sample size, follow-up duration, and post-transplant outcomes [4–9].

Multiple studies have indicated that the number of HT in patients with diabetes has recently increased or stabilized across different regions [10]. In Spain, a population-based study conducted between 2001 and 2015 found that the admission rates for HT declined over time among individuals with T2D (from 2.05 to 1.19 cases per 100,000 inhabitants; P < 0.001) [11]. Data on survival rates following HT in patients with diabetes remain inconsistent. Some studies report lower survival rates among HT recipients with diabetes compared to their non-diabetic counterparts [8], while other studies find similar rates in both groups or even higher among HT recipients without diabetes [6, 12–14]. Previous investigations in Spain have shown the effect of the COVID-19 pandemic on the use of heart procedures and hospital outcomes in people with diabetes [15, 16]. However, more studies are needed on HT in this population during the pandemic.

Therefore, the objectives of our investigation were as follows: (i) to evaluate temporal trends in incidence, clinical features, complications, and hospital outcomes among patients with T2D and non-diabetic patients who underwent HT in Spain from 2002 to 2021; (ii) to compare study variables between recipients with T2D and non-diabetic patients using propensity score matching (PSM); (iii) to identify variables associated with in-hospital mortality (IHM) among patients with and without T2D; and (iv) to ascertain the influence of T2D on IHM.

## Methods

### Study design, study population, and data assessment

To achieve the proposed objectives, we conducted an observational, retrospective, population-based study. The study period ran from January 1, 2002, to December 31, 2021. The study was based on the Spanish National Hospital Discharge Database (RAE-CMBD, *Registro de Actividad de Atención Especializada-Conjunto Mínimo Básico de Datos*), which collects individual patient information (e.g., sex, age, admission, and discharge date), diagnosis (1 to 20), and procedures (0 to 20), as well as the discharge destination (home, voluntary discharge, social institution, deceased). Diagnosis includes those conditions present at admission or diagnosed during hospitalization. Procedures include therapeutic or diagnostic procedures conducted during admission. The International Classification of Diseases Ninth Revision (ICD-9-CM) was used by the RAE-CMBD for coding from 2002 to 2015, and the Tenth Revision (ICD-10) was used from 2016 to 2021 [17]. All codes applied in this investigation are shown in Supplementary Table 1.

The study population included patients aged ≥ 35 years with an ICD-9 and ICD-10 code for HT in any position of the RAE-CMBD procedure fields. It was subsequently stratified according to diabetes status as T2D patients (ICD-9 codes: 250.x0 and 250.x2; ICD-10 codes: E11.x) and non-diabetic patients, in any diagnostic position. Patients with type 1 diabetes (T1D) codes (ICD-9 codes: 250.x1 and 250.x3; ICD-10 codes: E10.x) or missing data for essential variables (age, sex, date of admission or discharge, and discharge destination) were excluded. Recipients younger than 35 years were not included because the prevalence of Type 2 Diabetes (T2D) is extremely low in this age group in our country, so these patients may have Type 1 Diabetes (T1D) [18, 19]. For the purposes of this study, we also excluded all patients who underwent multi-organ transplantation (n = 77), including those who received both a heart and kidney transplant (n = 18), as well as those who underwent re-transplantation (n = 89).

### Study covariates

The study covariates collected for each HT recipient were age, sex and year of surgery. To achieve our objectives, the study years were grouped into five time periods, each consisting of four years (2002–2005, 2006–2009, 2010–2013, 2014–2017, and 2018–2021).

To assess the presence of comorbidity the Charlson Comorbidity Index (CCI) was used, excluding diabetes and heart diseases, based on a methodology described elsewhere for ICD based databases [20, 21]. The CCI is commonly used in epidemiological investigation to control the risk of in-hospital mortality according to 19 conditions present at the time of hospital admission. [20, 21]. The CCI was categorized in CCI = 0, CCI = 1 and CCI ≥ 2 and analyzed as a continuous variable.

Regardless of the diagnostic position in the database, comorbid conditions including obesity and pulmonary hypertension was assessed. The concomitant heart conditions potentially leading to HT analyzed were valvular heart disease, dilated cardiomyopathy, ischemic heart disease, and congenital heart disease. Data on complications of HT and the presence of pneumonia were also collected. Complications of HT included, rejection, failure, infection, cardiac allograft vasculopathy and “other” or “unspecified” complications of HT.

To identify possible infections caused by specific microorganisms, we searched the database for the following infections: *Staphylococcus* bacteremia, *Streptococcus* bacteremia, Gram-negative bacteremia, *Pseudomonas aeruginosa* infection, and cytomegalovirus infection. According to the RAE-CMBD methodology, only microorganisms confirmed through culture or positive results on genetic testing (PCR) can be recorded.

The use of hemodialysis, extracorporeal membrane oxygenation (ECMO), and tracheostomy were evaluated regardless of the position of procedures in the database.

The ICD9 and ICD10 codes for comorbidities, procedures and complications are shown in Supplementary Tables 1 and 2.

Length of hospital stay (LOHS) was defined as the number of days between admission and discharge. IHM was the proportion of deaths during the hospital admission for HT. Finally, the type of hospital admission, whether categorized as “Urgent” or “Programmed,“ was described and analyzed.

### Propensity score matching

The PSM method consisted of selecting for each patient with a T2D code a patient without a T2D code and with the same or closest propensity score (PS) obtained through multivariable logistic regression. We followed this approach to match the structure of the confounding factors for both groups. We used year of hospitalization, sex, age, and all comorbidities present on admission, including obesity and pulmonary hypertension, as matching conditions to calculate the PS [22].

The chosen matching method was one-to-one using calipers with a width equal to 0.2 of the standard deviation of the logit of the PS. Estimating the absolute standardized difference before and after matching enabled us to evaluate the quality of the PSM process. Populations are considered to be well balanced when the absolute standardized differences are < 10% after PSM [22]. A love plot was generated to visualize how populations become comparable after PSM. PSM has been previously used by other authors to compare the outcomes of HT according to clinical conditions and procedures [23–30].

### Statistical analysis

A statistical analysis was conducted to describe and compare the total number and covariates of patients who underwent HT (with T2D and without T2D). The results obtained in the descriptive analysis were expressed as frequencies and percentages for categorical variables and as mean and standard deviation or median with interquartile range (IQR) for quantitative variables.

The temporal trend was analyzed using the Cochran-Mantel-Haenszel statistic or Cochran-Armitage test for categorical variables and a linear regression *t* test or Jonckheere-Terpstra test for continuous variables.

Categorical variables were compared using the Fisher exact test. Continuous variables were compared using the *t* test or the Mann-Whitney test, as required.

In our investigation, we aim to test multiple hypotheses concurrently within a single study. This approach increases the risk of a Type I error, making it necessary to adjust the P-value accordingly [31]. To address this, we have employed the Bonferroni adjustment. As a result, the significance level must be divided by the number of hypothesis tests conducted. In each table, we have included a footnote specifying the P-value that should be considered significant after applying the Bonferroni adjustment.

We used multivariable Cox regression to identify the variables associated with IHM among patients who underwent HT according to diabetes status. Models were constructed including sex, age, year, comorbidities (CCI and all the specific clinical conditions analyzed), and obesity as covariates. The results for these models are shown with the hazard ratio (HR) and 95% confidence interval (CI).

We used Stata version 14 to perform the statistical analysis (Stata, College Station, TX, USA). Statistical significance was set at p < 0.05 (2-tailed).

### Sensitivity analysis

Using the entire database (patients with and without diabetes), we aimed to confirm the result of PSM for the effect of T2D on IHM. To do so we constructed a multivariable Cox regression model with IHM as the dependent variable adjusted for all the variables significantly associated with IHM in the bivariate analysis.

### Ethics statement

The RAE-CMBD is owned by the Spanish Ministry of Health and can be accessed upon request [32]. We sent the protocol for this investigation to the Ministry, which approved it and provided us with the anonymized database. According to Spanish legislation, written consent from the patients is not required, as this is an administrative registry.

## Results

Between the years 2002 and 2021, a total of 4429 HTs were performed in Spain. Of these, 19.14% (n = 848) were in patients with a code for T2D. The prevalence of T2D has remained stable over time, with values slightly below 20% (p = 0.388).

### Temporal trends in the number and characteristics of heart recipients according to diabetes status

As displayed in Table 1, there was a small increase in the number of HTs among recipients with T2D between 2002 and 2005 (n = 171) and 2018–2021 (n = 186). Women accounted for between 13% and 15.7% of patients, respectively. Over time, mean age increased (57.68 years in 2002–2005 vs. 59.27 years in 2018–2021; p = 0.025), as did comorbidity based on the mean CCI (p < 0.001), the frequency of obesity (7.6% vs. 12.37%; p = 0.020), and pulmonary hypertension (14.04% vs. 16.13%; p = 0.022).

**Table 1:** Clinical characteristics of hospital admissions for heart transplantation among patients with and without type 2 diabetes mellitus (T2D) in Spain, 2002–2021

Year	2002–2005	2006–2009	2010–2013	2014–2017	2018–2021	P trend
**T2D**						
N (%)*	171(19.19)	181(19.87)	154(17.43)	156(20.95)	186(19.15)	0.388
Women, n (%)	25(14.62)	28(15.47)	20(12.99)	21(13.46)	29(15.59)	0.947
Age, mean (SD)	57.68(6.13)	59.83(7.91)	58.34(6.8)	59.58(6.89)	59.27(7.17)	0.025
35–49 years, n (%)	15(8.77)	19(10.5)	18(11.69)	16(10.26)	19(10.22)	0.166
50–59 years, n (%)	82(47.95)	63(34.81)	65(42.21)	53(33.97)	65(34.95)	
≥ 60 years, n (%)	74(43.27)	99(54.7)	71(46.1)	87(55.77)	102(54.84)	
CCI, mean (SD)	1.49(0.81)	1.58(0.93)	1.64(0.87)	2.06(1.24)	2.28(0.92)	< 0.001
CCI = 0, n (%)	13(7.6)	16(8.84)	10(6.49)	10(6.41)	3(1.61)	< 0.001
CCI = 1, n (%)	83(48.54)	79(43.65)	61(39.61)	46(29.49)	30(16.13)	
CCI ≥ 2, n (%)	75(43.86)	86(47.51)	83(53.9)	100(64.1)	153(82.26)	
Obesity, n(%)	13(7.6)	6(3.31)	13(8.44)	9(5.77)	23(12.37)	0.020
Pulmonary hypertension, n(%)	24(14.04)	16(8.84)	33(21.43)	20(12.82)	30(16.13)	0.022
Valvular heart disease, n(%)	19(11.11)	22(12.15)	23(14.94)	28(17.95)	38(20.43)	0.081
Dilated cardiomyopathy, n(%)	104(60.82)	94(51.93)	96(62.34)	94(60.26)	123(66.13)	0.084
Ischemic heart disease, n(%)	101(59.06)	120(66.3)	79(51.3)	82(52.56)	97(52.15)	0.020
Congenital heart disease, n(%)	0(0)	0(0)	3(1.95)	4(2.56)	3(1.61)	0.097
**NO DIABETES**						
N	757	762	621	739	702	
Women, n (%)	126(16.64)	181(23.75)	159(25.6)	185(25.03)	189(26.92)	< 0.001
Age, mean (SD)	53.97(8.52)	55.64(9.22)	55.29(8.82)	54.97(9.26)	55.32(8.89)	0.004
35–49 years, n (%)	215(28.4)	187(24.54)	157(25.28)	206(27.88)	190(27.07)	0.008
50–59 years, n (%)	327(43.2)	295(38.71)	232(37.36)	274(37.08)	254(36.18)	
≥ 60 years, n (%)	215(28.4)	280(36.75)	232(37.36)	259(35.05)	258(36.75)	
CCI, mean (SD)	1.45(0.8)	1.49(0.83)	1.54(0.83)	1.77(0.94)	2.02(0.95)	< 0.001
CCI = 0, n (%)	59(7.79)	63(8.27)	37(5.96)	37(5.01)	22(3.13)	< 0.001
CCI = 1, n (%)	379(50.07)	353(46.33)	298(47.99)	274(37.08)	185(26.35)	
CCI ≥ 2, n (%)	319(42.14)	346(45.41)	286(46.05)	428(57.92)	495(70.51)	
Obesity, n(%)	19(2.51)	23(3.02)	20(3.22)	29(3.92)	39(5.56)	0.022
Pulmonary hypertension, n(%)	107(14.13)	110(14.44)	97(15.62)	96(12.99)	80(11.4)	0.208
Valvular heart disease, n(%)	125(16.51)	115(15.09)	107(17.23)	183(24.76)	166(23.65)	< 0.001
Dilated cardiomyopathy, n(%)	499(65.92)	486(63.78)	392(63.12)	462(62.52)	443(63.11)	0.689
Ischemic heart disease, n(%)	360(47.56)	351(46.06)	250(40.26)	301(40.73)	256(36.47)	< 0.001
Congenital heart disease, n(%)	12(1.59)	17(2.23)	18(2.9)	33(4.47)	43(6.13)	< 0.001

* N* number of admissions with heart transplantation, * Prevalence of T2D among patients who underwent a heart transplantation. *CCI* Charlson comorbidity index. The Charlson comorbidity index applies to different disease categories, the scores of which are added to obtain an overall score for each patient. We divided patients into three categories: low CCI (patients with no previously recorded disease), medium CCI (patients with one category), and high CCI (patients with two or more disease categories). To calculate the CCI, we used all disease categories, excluding diabetes and heart diseases. According to Bonferroni adjustment p values can be considered significant if < 0.0018 (0.05/28)

In patients with T2D, dilated cardiomyopathy was the heart condition most frequently coded (60.26%), followed by ischemic heart disease (56.49%). However, the frequency of ischemic heart disease decreased between 2002 and 2005 and 2018–2021 (59.06% vs. 52.15%; p = 0.020), and that of the other heart conditions remained stable.

Trends were similar in patients with and without T2D, although in the latter, higher values were recorded for the number of women undergoing HT (16.64% in 2002–2005 vs. 26.92% in 2018–2021; p < 0.001) and the frequency of valvular heart disease, ischemic heart disease, and congenital heart disease (all p < 0.001).

### Temporal trends in the prevalence of complications, procedures, and hospital outcomes among heart recipients according to diabetes status

As can been seen in Table 2, the proportion of recipients with T2D who experienced complications of HT increased significantly from 26.9% in 2002–2005 to 42.47% in 2018–2021 (p < 0.001). Significant increases over time were observed for the incidence of *Staphylococcus* bacteremia (2.92% vs. 10.22%; p < 0.001), Gram-negative bacteremia (2.92% vs. 4.84%; p < 0.001), and *Pseudomonas aeruginosa* (1.75% vs. 7.53%; p = 0.015). The procedures whose frequencies increased significantly over time were hemodialysis (5.85% vs. 17.74%; p < 0.001) and ECMO (0% vs. 17.74%; p < 0.001). IHM decreased, albeit not significantly, from 14.62 to 11.29% (p = 0.419), and the LOHS rose from a median of 24 days in 2002–2005 to 28 days in 2018–2021 (p < 0.001).

**Table 2:** Complications, isolate pathogens, procedures and outcomes of hospital admissions for heart transplantation among patients with and without type 2 diabetes (T2D) in Spain, 2002–2021

Year	2002–2005	2006–2009	2010–2013	2014–2017	2018–2021	P trend
**T2D**						
Complications of HT, n(%)	46(26.9)	39(21.55)	52(33.77)	48(30.77)	79(42.47)	< 0.001
Pneumonia, n (%)	5(2.92)	2(1.1)	3(1.95)	7(4.49)	10(5.38)	0.131
*Staphylococcus* bacteremia, n (%)	5(2.92)	4(2.21)	1(0.65)	9(5.77)	19(10.22)	< 0.001
*Streptococcus* bacteremia, n (%)	0(0)	0(0)	1(0.65)	2(1.28)	2(1.08)	0.390
Gram-negative bacteremia, n (%)	5(2.92)	8(4.42)	5(3.25)	21(13.46)	9(4.84)	< 0.001
*Pseudomonas aeruginosa*, n (%)	3(1.75)	4(2.21)	3(1.95)	7(4.49)	14(7.53)	0.015
Cytomegalovirus infection, n (%)	1(0.58)	4(2.21)	1(0.65)	6(3.85)	8(4.3)	0.065
Hemodialysis, n (%)	10(5.85)	8(4.42)	12(7.79)	19(12.18)	33(17.74)	< 0.001
ECMO, n (%)	0(0)	2(1.1)	8(5.19)	14(8.97)	33(17.74)	< 0.001
Tracheostomy, n (%)	4(2.34)	9(4.97)	7(4.55)	7(4.49)	11(5.91)	0.587
LOHS, median (IQR)	24(21)	22(22)	20(16)	28(27.5)	28(30)	< 0.001
IHM, n(%)	25(14.62)	23(12.71)	22(14.29)	13(8.33)	21(11.29)	0.419
**NO DIABETES**						
Complications of HT, n(%)	237(31.31)	215(28.22)	218(35.1)	245(33.15)	316(45.01)	< 0.001
Pneumonia, n (%)	41(5.42)	40(5.25)	46(7.41)	52(7.04)	63(8.97)	0.028
*Staphylococcus* bacteremia, n (%)	46(6.08)	43(5.64)	49(7.89)	49(6.63)	61(8.69)	0.127
*Streptococcus* bacteremia, n (%)	5(0.66)	1(0.13)	3(0.48)	3(0.41)	3(0.43)	0.620
Gram-negative bacteremia, n (%)	26(3.43)	35(4.59)	63(10.14)	98(13.26)	78(11.11)	< 0.001
*Pseudomonas aeruginosa*, n (%)	13(1.72)	17(2.23)	30(4.83)	32(4.33)	39(5.56)	< 0.001
Cytomegalovirus infection, n (%)	16(2.11)	20(2.62)	26(4.19)	41(5.55)	53(7.55)	< 0.001
Hemodialysis, n (%)	67(8.85)	84(11.02)	97(15.62)	141(19.08)	158(22.51)	< 0.001
ECMO, n (%)	2(0.26)	18(2.36)	54(8.7)	126(17.05)	164(23.36)	< 0.001
Tracheostomy, n (%)	29(3.83)	55(7.22)	60(9.66)	76(10.28)	83(11.82)	< 0.001
LOHS, median (IQR)	26(26)	25.5(31)	28(35)	33(42)	33.5(44)	
IHM, n(%)	130(17.17)	158(20.73)	124(19.97)	125(16.91)	108(15.38)	0.046

*HT* heart transplantation. *ECMO* extracorporeal membrane oxygenation. *LOHS* length of hospital stay; *IQR*: Interquartile range; *IHM*: In-hospital mortality. According to Bonferroni adjustment p values can be considered significant if < 0.0018 (0.05/28)

Regarding non-diabetic patients, the percentage of those with complications recorded in the discharge report increased significantly (p < 0.001), as did the presence of pneumonia and pathogens such as Gram-negative microorganisms, *Pseudomonas aeruginosa*, and cytomegalovirus (all p < 0.001). The frequency of hemodialysis, ECMO, and tracheostomy also increased significantly (all p < 0.001).

In non-T2D recipients, LOHS increased over time (26 days in 2002–2005 vs. 33 days in 2018–2021; p < 0.001). However, the IHM decreased from 17.17% in 2002–2005 to 15.38% in 2018–2021 (non-significant, p = 0.248).

### Comparison of characteristics, clinical variables, and hospital outcomes between heart recipients according to diabetes status

Before PSM, when all patients hospitalized from 2002 to 2021 were grouped (see Table 3), there were more women in the non-diabetic group than in the T2D group (23.22% vs. 14.74%; p < 0.001). However, recipients with T2D were significantly older than non-T2D patients (58.91 years vs. 54.99 years; p < 0.001) and had more comorbid conditions according to the mean CCI (1.8 vs. 1.63; p < 0.001). Specifically, recipients with T2D more frequently had peripheral vascular disease and kidney disease (21.37% and 18.45% vs. 16.22% and 10.22%, respectively). Moreover, recipients with T2D were more frequently obese (7.42% vs. 3.51%; p < 0.001) and had ischemic heart disease (56.92% vs. 43.06%; p < 0.001). Nonetheless, non-T2D patients more frequently had valvular heart disease, congenital heart disease, complications of HT, pneumonia, *Staphylococcus* bacteremia, Gram-negative bacteremia, and cytomegalovirus infection. After PSM, the differences observed between patients with and without T2D became non-significant, except for pneumonia (6.97% in the non-diabetic group vs. 3.04% in the T2D group; p < 0.001) and Gram-negative bacteremia (7.87% in the non-diabetic group vs. 5.4% in the T2D group; p = 0.036).

**Table 3:** Comparison of characteristic, comorbidities, transplant complications and hospital outcomes among patients with and without type 2 diabetes (T2D) who underwent a heart transplantation in Spain from 2002 to 2021, before and after propensity score matching (PSM)

	Before PSM			After PSM		
	T2D	No diabetes	P	T2D	No diabetes	P
N	889	3816	NA	889	889	NA
Year 2202 − 2005, n(%)	171(20.17)	757(21.14)	0.388	171(20.17)	171(20.45)	0.362
Year 2006–2009, n(%)	181(21.34)	762(21.28)		181(21.34)	185(22.13)	
Year 2010–2013, n(%)	154(18.16)	621(17.34)		154(18.16)	131(15.67)	
Year 2014–2017, n(%)	156(18.4)	739(20.64)		156(18.4)	180(21.53)	
Year 2018–2021, n(%)	186(21.93)	702(19.6)		186(21.93)	169(20.22)	
Women, n (%)	131(14.74)	886(23.22)	< 0.001	131(14.74)	116(13.05)	0.304
Age, mean (SD)	58.91(7.02)	54.99(8.93)	< 0.001	58.91(7.02)	59.19(7.69)	0.421
35–49 years, n (%)	90(10.12)	1016(26.62)	< 0.001	90(10.12)	98(11.02)	0.601
50–59 years, n (%)	347(39.03)	1487(38.97)		347(39.03)	328(36.9)	
≥ 60 years, n (%)	452(50.84)	1313(34.41)		452(50.84)	463(52.08)	
CCI, mean (SD)	1.8(1)	1.63(0.89)	< 0.001	1.80(1.00)	1.75(0.99)	0.390
Peripheral vascular disease, n (%)	190(21.37)	619(16.22)	< 0.001	190(21.37)	174(19.57)	0.347
Cerebrovascular disease, n (%)	51(5.74)	196(5.14)	0.470	51(5.74)	47(5.29)	0.678
Dementia, n (%)	0(0)	2(0.05)	0.495	0(0)	1(0.11)	0.317
Chronic respiratory disease, n (%)	153(17.21)	648(16.98)	0.870	153(17.21)	152(17.1)	0.950
Rheumatoid disease, n (%)	3(0.34)	35(0.92)	0.082	3(0.34)	3(0.34)	1.000
Peptic ulcer, n (%)	5(0.56)	37(0.97)	0.245	5(0.56)	3(0.34)	0.479
Mild liver disease, n (%)	35(3.94)	206(5.4)	0.075	35(3.94)	34(3.82)	0.902
Hemiplegia or paraplegia, n (%)	8(0.9)	52(1.36)	0.268	8(0.9)	7(0.79)	0.795
Renal disease, n (%)	164(18.45)	390(10.22)	< 0.001	164(18.45)	141(15.86)	0.148
Cancer, n (%)	3(0.34)	20(0.52)	0.472	3(0.34)	6(0.67)	0.316
Moderate/Severe liver disease, n (%)	12(1.35)	33(0.86)	0.181	12(1.35)	11(1.24)	0.834
AIDS, n (%)	0(0)	2(0.05)	0.495	0(0)	1(0.11)	0.317
Obesity, n(%)	66(7.42)	134(3.51)	< 0.001	66(7.42)	50(5.62)	0.124
Pulmonary hypertension, n(%)	126(14.17)	516(13.52)	0.610	126(14.17)	116(13.05)	0.489
Valvular heart disease, n(%)	131(14.74)	731(19.16)	0.002	131(14.74)	117(13.16)	0.338
Dilated cardiomyopathy, n(%)	541(60.85)	2427(63.6)	0.127	541(60.85)	542(60.97)	0.961
Ischemic heart disease, n(%)	506(56.92)	1643(43.06)	< 0.001	506(56.92)	531(59.73)	0.229
Congenital heart disease, n(%)	10(1.12)	123(3.22)	0.001	10(1.12)	11(1.24)	0.826
Complications of HT, n(%)	268(30.15)	1293(33.88)	0.033	268(30.15)	301(33.86)	0.093
Pneumonia, n (%)	27(3.04)	256(6.71)	< 0.001	27(3.04)	62(6.97)	< 0.001
*Staphylococcus* bacteremia, n (%)	41(4.61)	259(6.79)	0.017	41(4.61)	58(6.52)	0.079
*Streptococcus* bacteremia, n (%)	5(0.56)	15(0.39)	0.485	5(0.56)	3(0.34)	0.479
Gram-negative bacteremia, n (%)	48(5.4)	308(8.07)	0.007	48(5.4)	70(7.87)	0.036
*Pseudomonas aeruginosa*, n (%)	31(3.49)	132(3.46)	0.967	31(3.49)	31(3.49)	1.000
Cytomegalovirus infection, n (%)	20(2.25)	161(4.22)	0.006	20(2.25)	26(2.92)	0.370
Hemodialysis, n (%)	84(9.45)	564(14.78)	< 0.001	84(9.45)	122(13.72)	0.005
ECMO, n (%)	57(6.41)	364(9.54)	0.003	57(6.41)	89(10.01)	0.006
Tracheostomy, n (%)	39(4.39)	315(8.25)	< 0.001	39(4.39)	62(6.97)	< 0.001
LOHS, median (IQR)	23(24)	28(36)	< 0.001	23(24)	28(36)	< 0.001
IHM, n(%)	109(12.26)	693(18.16)	< 0.001	109(12.26)	163(18.34)	0.018
Urgent hospital admission n(%)	542(60.97)	1944(50.94)	< 0.001	542(60.97)	456(51.29)	< 0.001

HT heart transplantation ECMO extracorporeal membrane oxygenation LOHS: length of hospital stay; IQR: Interquartile range; IHM: In-hospital mortality. *CCI* Charlson comorbidity index.NA: not available. According to Bonferroni adjustment p values can be considered significant if < 0.0014 (0.05/36)

After PSM, recipients with T2D less frequently required hemodialysis (9.45% vs. 13.72%; p = 0.005), ECMO (6.41% vs. 10.01%; p = 0.006), and tracheostomy (4.39% vs. 6.97%; p < 0.001) than non-T2D patients.

The median LOHS was 28 days for non-T2D patients and 23 days for recipients with T2D (p < 0.001). IHM among non-T2D patients remained over 4% higher than among recipients with T2D (18.34% vs. 12.26%; p = 0.018).

The proportion of recipients with Type 2 Diabetes (T2D) who were coded for “Urgent” admission was significantly higher than that of those without diabetes (60.97% vs. 50.94%; p < 0.001).

**Fig. 1:**
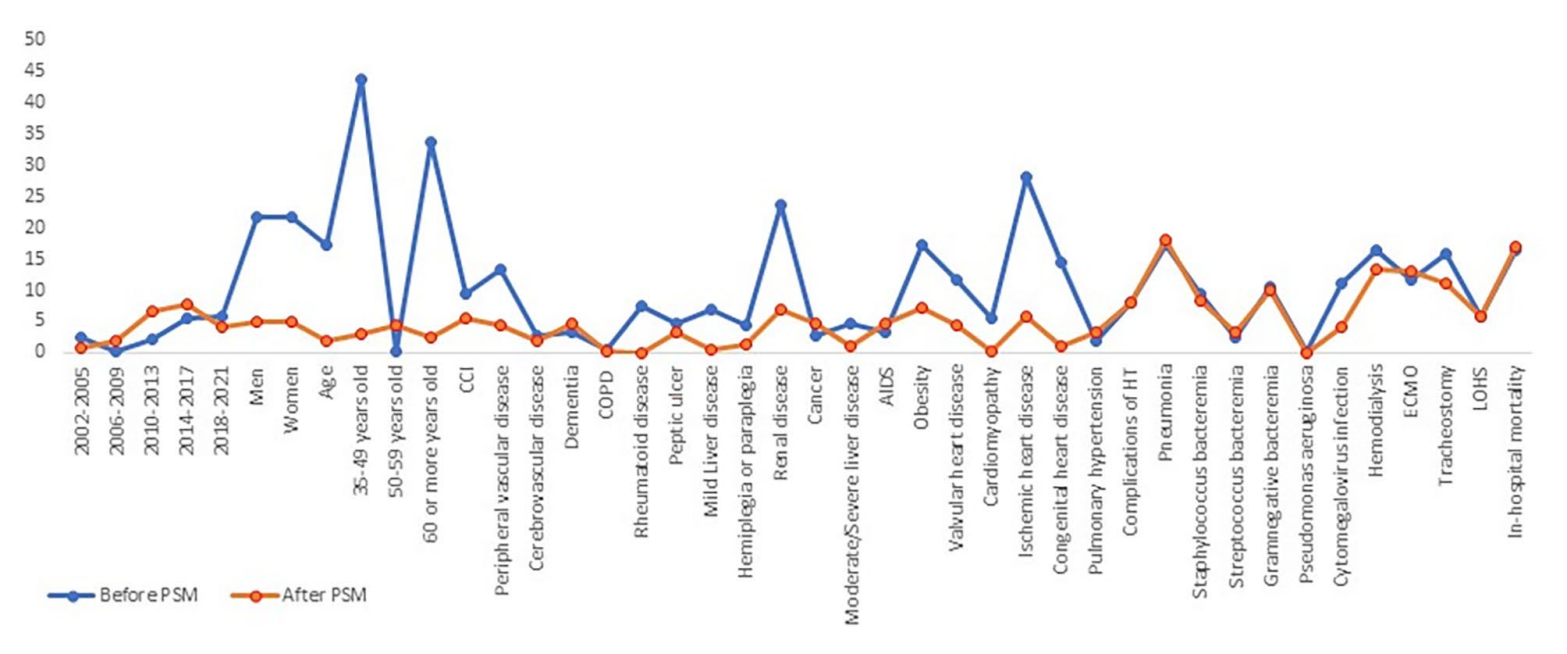
Love plot showing the comparison of covariate values for patients with and without diabetes: absolute standardized differences before and after propensity score matching (PSM). *Footnote: CCI* Charlson comorbidity index. COPD Chronic obstructive pulmonary disease. HT Heart transplantation ECMO extracorporeal membrane oxygenation LOHS: length of hospital stay;

Figure 1 shows the absolute standardized differences before and after PSM. A significant imbalance could be ruled out, since the absolute standardized differences after PSM were below 10% for all the variables included in the PS [22].

### Variables associated with in-hospital mortality among heart recipients according to diabetes status

Table 4 shows the results of the multivariable Cox regression analysis performed to identify variables associated with IHM after HT in patients with T2D, without T2D, and for all patients who underwent this procedure.

**Table 4:** Multivariable Cox regression analysis of the factors associated with mortality during hospital admission for heart transplantation in Spain, 2002–2021 according to diabetes status

	T2D	No diabetes	ALL
	HR (95% CI)	HR (95% CI)	HR (95% CI)
Year 2002–2005	1	1	1
Year 2006–2009	1.06(0.59–1.92)	1.13(0.89–1.43)	1.1(0.89–1.37)
Year 2010–2013	0.81(0.43–1.55)	0.88(0.68–1.14)	0.88(0.69–1.11)
Year 2014–2017	0.4(0.19–0.86)	0.6(0.46–0.79)	0.57(0.45–0.73)
Year 2018–2021	0.38(0.19–0.77)	0.41(0.3–0.55)	0.41(0.31–0.53)
Age, 35–49 years	1	1	1
Age, 50–59 years	2.08(0.85–5.05)	1.11(0.89–1.37)	1.16(0.95–1.43)
Age, ≥ 60 years	2.53(1.04–5.95)	1.6(1.29–1.98)	1.61(1.32–1.98)
Hemodialysis	2.47(1.46–4.16)	2.02(1.7–2.41)	2.07(1.76–2.44)
ECMO	3.12(1.66–5.87)	2.24(1.8–2.8)	2.34(1.9–2.88)
Complications of HT	1.72 (1.07–4.98)	2.35(1.84–2.98)	1.97(1.42–2.56)
T2D	NA	NA	0.77(0.63–0.98)

Calculated using Cox regression models: HR: Hazard Ratio. CI Confidence interval. HT heart transplantation ECMO extracorporeal membrane oxygenation *T2D* Type 2 diabetes. NA: not available

For all three populations analyzed, the variable associated with lower IHM was more recent surgery; those associated with a higher IHM were older age, presenting complications of HT, and hemodialysis or ECMO during hospitalization.

The results of the sensitivity analysis confirmed those found after PSM. As can be seen in Table 4, when all patients hospitalized from 2002 to 2021 were included in the Cox multivariable model, the presence of T2D was associated with a lower IHM after HT (HR 0.77; 95%CI 0.63–0.98).

## Discussion

The findings of this nationwide retrospective study, which is based on the diabetes status of over 4400 patients who underwent HT in Spain between 2002 and 2021, revealed several significant insights. First, a slight increase in the number of HTs among patients with T2D was observed when the last period (2018–2021) was compared with the first one (2002–2005). Second, IHM has decreased over the last 20 years for individuals with and without T2D. Third, IHM after HT was lower in patients with T2D than in non-diabetic individuals after adjustment based on PSM and multivariable Cox regression analysis.

Consistent with de Miguel-Yanes et al., we recorded a decrease in the number of HT procedures in patients with T2D from 2002 to 2005 to 2014–2017 [11]. Nevertheless, despite the overall decrease in transplants reported during the COVID-19 pandemic, a slight increase was observed in the last period of the study (2018–2021) [33, 34]. We posit that this result is a consequence of the increasing age of patients on the waiting lists for HT [35, 36] and that this increased age and comorbidity may have escalated the need for HT. Future studies are required to confirm this hypothesis.

The frequency of complications of HT rose in patients with and without diabetes between 2002 and 2005 and 2018–2021. Furthermore, we observed an increase in microorganisms causing bacteremia, such as *Staphylococcus*, Gram-negative bacteria, and *Pseudomonas aeruginosa*, and in the use of procedures such as hemodialysis and ECMO. This can be attributed to the high comorbidity typical of transplant recipients, immunosuppressive therapy, kidney disease, and hypertension [37], resulting in increased hospital stay in both groups in our study. Nevertheless, despite the higher number of complications and infections, we witnessed a significant reduction in IHM over the study period. Data from the Spanish Heart Transplant Registry for the general population also show a progressive improvement in HT outcomes in terms of survival in recent years, likely due to better control of primary graft failure and acute rejection [38].

Several epidemiological studies had previously indicated that recipients with T2D undergoing HT differ from other types of patients [7]. A recent study reported that patients with diabetes experienced poorer graft survival following transplantation (HR 1.17; 95% CI 1.08–1.26, P < 0.001), although the authors scored graft survival to further stratify the risk of patients with diabetes and found that graft survival was similar in low-risk diabetic patients and non-diabetic patients (HR 0.91; 95% CI 0.82–1.01) [23]. In our study, individuals with T2D were less likely to have pneumonia and Gram-negative bacterial infections or undergo hemodialysis, ECMO, and tracheostomy procedures. They also had a lower LOHS and lower IHM. This suggests that, owing to their pre-existing conditions at the time of transplantation, patients with diabetes have a lower risk of reduced graft survival [6] or have well-controlled postoperative blood sugar [39], although we do not have sufficient data to substantiate this argument.

As expected, after multivariable adjustments, we discovered that the use of hemodialysis and ECMO were risk factors for mortality in T2D patients who underwent HT. Shoji et al. [40] found that post-transplant dialysis patients had a higher risk of all-cause mortality (adjusted hazard ratio [aHR]: 5.2, 95% CI: 4.7–5.7, p < 0.001), and Lim et al. [41] recently observed that patients requiring ECMO as a bridge to HT were characterized by higher rates of preoperative multi-organ failure and early mortality than those who were extubated.

In line with previously published results [11, 42], and despite the fact that scores predicting early survival following HT do not consider diabetes a significant factor compared to other, more significant clinical factors [43], we found that the presence of T2D was associated with a lower risk of IHM during admission for HT. A key explanation for our results is that they likely reflect an improvement in both pre-transplant and post-transplant management of diabetes and vascular risk factors [6, 44].

The strengths of our study include the use of a national population database (RAE-CMBD) over a 20-year period and a methodology that has been used elsewhere [11]. Furthermore, the RAE-CMBD collects virtually all hospitalizations in Spain (> 95%) and our data are consistent with those published by the National Transplant Organization of Spain [45].

Our study is subject to a series of limitations. First, like other observational and retrospective studies that rely on ICD codes from discharge databases, our investigation may be affected by low sensitivity and specificity. This can depend on the physicians’ proficiency in coding hospital procedures and diagnoses, which can, in turn, influence data quality. Coding is performed for administrative rather than research purposes, primarily focusing on economic considerations. Second, our study does not consider clinical or performance parameters and cannot assess events that may occur in patients after hospital discharge. Third, ICD codes do not provide information on disease severity, functional status, the type of T2D treatment (e.g., insulin or antidiabetic drugs), or the reasoning behind the intensity of the treatment provided. Fourth, in the RAE-CMBD database, the number of ICD codes that physicians can record is limited to 20 diagnoses and 20 procedures per patient. Additionally, the cause of death is not specified in the database, and sociodemographic variables such as race, education level, and social class are not collected. Fifth, our study excluded patients who had undergone multi-organ transplantation and re-transplantation. However, this represents a very low proportion of HT patients (< 4%), as multi-organ transplantation and re-transplantation are rare procedures in our country [46]. In our opinion, excluding these cases would have minimal impact on our results but should be considered for future investigations. Finally, ICD codes in the database do not consider donor demographics and clinical conditions. As a result, we could not incorporate variables describing recipient, donor, and transplant factors, including body mass index (BMI), peak panel reactive antibodies, human leukocyte antigen mismatches, cold ischemia time, immunosuppressant therapy, induction therapy, mechanical circulatory support devices used before surgery, previous sternotomies, and clinical factors such as wound or sternotomy infections, delayed graft function, or episodes of rejection.

Despite these limitations, hospital discharge databases based on ICD codes have been used widely in epidemiological research by many authors from various countries. Such databases have proven useful for studying temporal trends as well as the characteristics, procedures, and hospital outcomes of HT recipients [9, 11, 37, 47–50].

## Conclusions

In conclusion, between 2002 and 2017, the number of HT procedures in Spain decreased in patients with T2D but increased slightly in the last study period (2018–2021). Lower values were recorded in recipients with T2D than in non-T2D patients for presence of pneumonia and Gram-negative bacteremia and for the use of procedures such as hemodialysis, ECMO, and tracheostomy. In addition, LOHS and IHM were lower in recipients with T2D. The main risk factors for IHM following HT in recipients with T2D were complications of HT and the need for hemodialysis and ECMO. IHM following a heart transplant has decreased in Spain in individuals with and without T2D. Finally, the presence of T2D was associated with a lower risk of IHM than in non-diabetic patients.

### Electronic supplementary material

Below is the link to the electronic supplementary material.

Supplementary Material 1

## Data Availability

According to the contract signed with the Spanish Ministry of Health, which provided access to the databases from the RAE-CMBD, we cannot share the databases with any other investigator, and we have to destroy the databases once the investigation has concluded. Consequently, we cannot upload the databases to any public repository. However, any investigator can apply for access to the databases by filling out the questionnaire available at http://www.msssi.gob.es/estadEstudios/estadisticas/estadisticas/estMinisterio/SolicitudCMBDdocs/Formulario_Peticion_Datos_CMBD.pdf. All other relevant data are included in the paper.

## Abbreviations

CCI: Charlson Comorbidity Index
ECMO: Extracorporeal membrane oxygenation
HT: Heart transplantation
ICD-9-CM: International Classification of Diseases Ninth Revision
ICD-10: International Classification of Diseases Tenth Revision
IHM: In-hospital mortality
LOHS: Length of hospital stay
PS: Propensity score
PSM: Propensity score matching
RAE-CMBD: Spanish National Hospital Discharge Database
T2D: Type 2 diabetes
