# Supplementary material for: Temporal trends and outcomes of heart transplantation in Spain (2002–2021): propensity score matching analysis to compare patients with and without type 2 diabetes
Source: Cardiovasc Diabetol. 2023 Sep 29;22:266. doi: 10.1186/s12933-023-01995-1 (PMC10542663; doi:10.1186/s12933-023-01995-1)
Supplement: Supplementary file 1 — Supplementary Material 1 [file 12933_2023_1995_MOESM1_ESM.docx]

**Supplementary Table 1. International Classification of Diseases Ninth Revision (ICD-9) and Tenth Revision (ICD-10) codes used in this investigation.**

| Variable | ICD-9 | ICD10 |
| --- | --- | --- |
| Heart transplantation | 37.51 | 02YA0Zx |
| Type 2 diabetes | 250·x0; 250·x2 | E11.x |
| Obesity | 278.00, 278.01, 278.02, 278.03 | E66.09, E66.1 E66.3, E66.8 E66.9 E66.2, E66.01 |
| Pulmonary hypertension | 416.0, 416.8 | I27.0, I27.2x, I27.89 |
| Valvular heart disease | 424.0-424.9x | I34-I37 |
| Cardiomyopathy | 394-397 | I05-I09 |
| Ischemic heart disease | 425.xx | I42.xx |
| Congenital heart disease | 410-414 | I20-I25 |
| Complications of heart transplantation | 996.83 | T86.2, T86.20, T86.21, T86.22, T86.23, T86.29, T86.290, T86.298 |
| Pneumonia | 481-486, 997.31 | J13-J18, J95.851 |
| *Staphylococcus* bacteremia | 038.10, 038.11, 038.12, 038.19, 041.10, 041.11,  041.12, 041.19 | A4101, A4102, A411, A412, A4901, A4902, B9561, B9562, B957, B958 |
| *Streptococcus* bacteremia | 038.0, 038.2, 041.00, 041.01, 041.02, 041.09 | A40.0, A40.1, A40.3, A40.8, A40.9, A49.1, B95.0, B95.1, B95.3, B95.4, B95.5 |
| Gram-negative bacteremia | 038.40, 038.42, 038.43, 038.44, 038.49, 041.3, 041.41, 041.42, 041.43, 041.49, 041.5, 041.6, 041.7 | A41.3, A41.50, A41.51, A41.52, A41.53, A41.59, B96.1, B96.20, B96.21, B96.22, B96.23, B96.29, B96.3, B96.4, B965 |
| *Pseudomonas aeruginosa* | 041.7 | B96.5 |
| Cytomegalovirus infection | 078.5, 484.1 | B25.x |
| Hemodialysis | 39.95, V56.0 | 5A1Dxxx |
| Extracorporeal membrane oxygenation | 39.65 | 5A15xxx |
| Tracheostomy | 31.1, 31.29 | 0B11xxx |

**Supplementary Table 2.** List of International Classification of Diseases Ninth Revision (ICD-9) and Tenth Revision (ICD-10) codes used to identify complications of heart transplant in this investigation.

| Variable | ICD-9 | ICD10 |
| --- | --- | --- |
| Complications of heart transplant | 996.83 | - |
| Heart transplant rejection |  | T86.21 |
| Heart transplant failure |  | T86.22 |
| Heart transplant infection |  | T86.23 |
| Cardiac allograft vasculopathy |  | T86.290 |
| Other or unspecified complications of heart transplant |  | T86.20, T86.29, T86.298 |
